# Testing ActiveYou II: Applying Cognitive Interviews in Improving Item Quality and Applicability of a Web-Based, Self-Report Instrument on Participation in Children with Disabilities

**DOI:** 10.3390/ijerph18094768

**Published:** 2021-04-29

**Authors:** Friedolin Steinhardt, Reidun Jahnsen, Anne-Stine Dolva, Anna Ullenhag

**Affiliations:** 1 Faculty of Social and Health Sciences, Inland Norway University of Applied Sciences, 2418 Elverum, Norway; anne-stine.dolva@inn.no; 2 Beitostølen Healthsports Center, 2953 Beitostølen, Norway; reijah@ous-hf.no (R.J.); anna.ullenhag@mdh.se (A.U.); 3 Research Center of Habilitation and Rehabilitation Models and Services, University of Oslo, 0315 Oslo, Norway; 4 Department of Health, Care and Social Welfare, Mälardalens University, 722220 Västerås, Sweden

**Keywords:** children with disabilities, participation, instrument development, cognitive interviews, self-reported, rehabilitation

## Abstract

Background: Children and youth with disabilities participate less in leisure activities than their nondisabled peers. Increasing participation is a primary goal of rehabilitation interventions. However, valid measures that include the individual’s perspectives and facilitating and hindering factors for participation are lacking in the Norwegian setting. In this study, ActiveYou II, a self-report, web-based instrument under development, was tested to obtain item quality and applicability. Methods: Nine children with disabilities participated in cognitive interviews, testing a first set of ActiveYou II items. The verbal probe method for cognitive interviews was applied. Results: The children’s comprehension and responses through cognitive interviews improved the applicability and item quality of ActiveYou II. Item adjustments were made to the wording of the questions and response alternatives, and the number of response alternatives were decreased where appropriate. Conclusions: The use of cognitive interviews with children before performing further psychometric testing has been very useful in the development process of ActiveYou II. Adjustments of the questions and response alternatives were made accordingly.

## 1. Introduction

Increasing participation in leisure activities is one of the major goals and outcomes of rehabilitation interventions for children and youth with disabilities [1,2,3]. This is grounded in an understanding of the positive effects of participation in leisure activities for the physical, emotional, and social development of children and their general well-being [4,5,6]. Valid and reliable instruments are needed to gain an increased understanding and knowledge of the participation patterns in leisure activities shown by children with disabilities and for planning and evaluating interventions [6,7]. In their systematic review, Adair et al. [6] argued that measures continually need to be adapted to be in accordance with the developing understanding of the participation construct. Furthermore, they promote bringing more of the individual’s subjective perspectives on participation into measures. Moreover, measures need to be appropriate for specific settings and regions, since factors influencing participation may vary between national and international settings [8,9].

To date, there is no valid web-based measure of participation for the Norwegian setting based on the child’s self-report, nor instruments that measure facilitating and hindering factors for participation. Prior studies have tested and validated Norwegian versions of Preferences for Activities for Children (PAC) and Children’s Assessment of Participation and Enjoyment (CAPE) [10,11]. However, the activities and items did not quite fit the Norwegian setting. There were also difficulties in administering the instruments, especially for children with intellectual disabilities [10]. Most crucially, the publisher of CAPE and PAC declined to publish the Norwegian versions due to the small market. Participants also wished for questionnaires that could be administered digitally [10]. Therefore, the self-reported and web-based questionnaire ActiveYou I (Norwegian: AktiveDeg I) was developed [12]. ActiveYou I measures a child’s activity preferences for participation in 17 different physical leisure activities [12]. At present, the companion measure, ActiveYou II (Norwegian: AktiveDeg II), is under development. ActiveYou II aims to evaluate current patterns of participation in different physical leisure activities, including individual experience and perceived facilitating and hindering factors. Both instruments are intended to be used to plan and evaluate rehabilitation interventions, focusing on participation in adapted physical activities [12]. However, both ActiveYou I and II are intended to be generic, and the activities can be changed according to different contexts and target groups [12].

In the International Classification of Functioning, Disability and Health (ICF), participation is defined as “involvement in life situations” [13]. However, there has been criticism of the definition given in the ICF, mainly regarding the lack of subjective perspectives on participation and the lack of clarity in the distinction between activity and participation [3,14]. Based on previous theoretical work, such as the family of Participation Related Construct model (fPRC) by Imms et al. [3], participation is defined as a multidimensional construct that describes both observable (objective) and unobservable (subjective) components that contribute to a person’s participation in life situations. According to King et al. [15], context includes personal, family, and environmental factors. Observable components are mainly attendance, participation frequency, diversity, and the social and physical context. Unobservable components are the individual’s cognitive (self-regulation, relevance for future endeavors, personal goals, autonomy) and/or affective engagement (feelings of identification and/or belonging, in relationship with adults and peers) [16]. This is the theoretical fundament of the development of ActiveYou II.

The aim of this study was to evaluate the item quality and applicability of ActiveYou II. The study explored the following questions: (i) Can cognitive interviews with children and youth (target group) improve item quality and applicability of ActiveYou II? (ii) Which adjustments are needed before advancing in the development process of ActiveYou II?

## 2. Methods

### 2.1. Participants and Procedure

Participants in the cognitive interviews were nine children within the target-group recruited from a rehabilitation center in Norway. The inclusion criteria were (1) school age (6–17), (2) ability to give informed consent, and (3) having the ability to understand and to communicate verbally and participate actively in the interviews in Norwegian. Thus, children with moderate/severe intellectual disability were excluded. Since ActiveYou II was supposed to be an instrument for a wide target group, it was deemed to have a sample of participants that represent different parts of this target-group.

Recruitment and data collection took place in November 2018. Children and their parents were informed about the purpose of the study, and information and consent forms were handed out to potential participants. Participation in the interviews was voluntary and had no effect on the participants’ intervention at the center. Parents could attend the interviews to observe or assist their children if they wished.

The verbal probe method was used, as recommended by Spencer et al. [17]. The first and last author designed an interview guide based on questions and formulations they expected would be difficult for the children. Interviews took place in small groups of a maximum of five children and lasted approximately 30 min. During the interviews, the questions being discussed were shown via a projector. The children were asked about general phrasing and understanding of the items and design, and the children were asked to identify individual words that seemed strange to them. The children were also asked to express themselves openly if any other part of the questionnaire seemed confusing or difficult to understand. In addition, all children received a paper copy of the questions, on which they could note their answers. The interviews were recorded through both a voice recorder and video. Potential issues of privacy and ethics were approved by the Norwegian Centre for Research Data (reference number 52305/3/STM).

### 2.2. ActiveYou II

**Figure 1:**
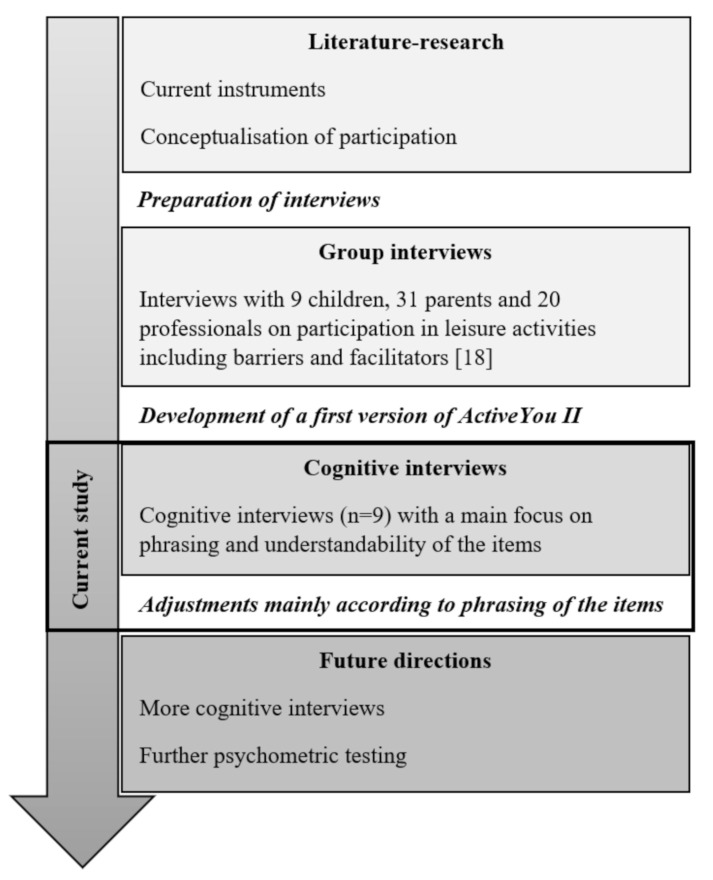
Flow-chart of the whole development process of ActiveYou II.

ActiveYou II aims to be a self-reported, web-based instrument to capture children’s and youths’ patterns of participation in physical activities. Figure 1 gives an overview of the whole development process of ActiveYou II thus far. In the process of developing ActiveYou II, group interviews with children, parents, and professionals were conducted to identify important facilitators and barriers for participation in leisure activities that should be included in the instrument [18]. Since ActiveYou II is supposed to be self-reported, it was deemed important to include the perspective of children and youth in the interview process. Children and youth with disabilities are expected to administer it themselves or with assistance from a caregiver. To answer, participants log into a password-protected homepage. The questionnaire can be administered from any device that supports standard internet browser applications. The 17 activities included are: pool activity, cross-country skiing, horseback riding, training in a fitness room/center, downhill skiing, climbing, outdoor activities, water activities outdoors, playing in the snow, going for a walk/hiking, gaming for training (e.g., Happy Rehab, Wii Sports, Let’s Dance…), rolling activities, move to music, group activities, play outside, cycling, individual activities. All activities are visualized for the children using a short slideshow of three photos that show the activity at hand with different performance modes with and without assistive activity devices. For each activity, children are asked about (1) their frequency of attendance, (2) with whom they participate, (3) their sense of mastery, (4) their level of involvement/attraction, (5) facilitating personal, familial, and environmental factors, and (6) hindering personal, familial, and environmental factors, before moving to the next activity.

Table 1 gives an overview of the items and the amendments made during the different phases of the study. Sense of mastery is defined as “the extent to which one regards one’s life chances as being under one’s own control” [19]. Involvement is defined in line with leisure research as “an unobservable state of motivation, arousal, or interest with respect to a recreational activity or associated product” [20] The items covering involvement in the questionnaire (e.g., Is it fun to do the activity? The activity is important for me) were taken from the Modified Involvement Scale designed by Kyle et al. [21], specifically the dimension of attraction, which includes the individual’s perceived importance, preference, and pleasure towards a specific activity [21].

### 2.3. Cognitive Interviews

The method is a form of qualitative data collection, in which the researcher goes through the instrument under development with an individual or group of participants for whom the instrument is designed. It is a verbal response method on items with the purpose of enhancing the understanding and meaning of the instrument [22]. During the interview, the researcher asks the informant(s) to speak out their thoughts on the phrasing of the items, their understanding of the meaning of the items, and their approach to responding [17,22]. According to Spencer, Bouffard, and Watkinson [17], this method is closely based on Tourangeau’s question-and-answer model developed in cognitive psychology [23]. Based on this theory, the individual has to complete four actions to answer the questions: (1) comprehension, which involves the individual’s understanding of the question; (2) retrieval, which involves the individual accessing memories and information relevant for the question; (3) judgment, which involves forming an answer to the given question based on the retrieval; and (4) response, which refers to the process of relating one’s answer to the given response categories.

This method is helpful in identifying discrepancies within the instrument in order to make adjustments for the targeted population before proceeding with further testing on larger samples [17,24]. There are two different approaches to cognitive interviews—the “think aloud” and “verbal probe” methods [22]. Using the think aloud method, the participant freely expresses his thought-process while answering the questionnaire, and the researcher takes a more passive/observant position. On the other hand, in the verbal probe method, the researcher takes a more active position by asking the participants about specific aspects of the questionnaire based on a previously designed interview-guide.

Cognitive interviews have shown to be efficient in instrument development [17,24]. When applying cognitive interviews with children, the verbal probe method is recommended [17]. Within healthcare and rehabilitation, Liljenquist et al. [25] described how the development of the Participation Experience Survey (PES) benefited from implementing cognitive interviews in the process of measure development. Spencer, Bouffard, and Watkinson [17] have discussed and tested cognitive interviews with children with disabilities in a setting focused around adapted physical activities. They used cognitive interviews to validate established instruments on the individual’s perception about their own athletic performance. In the development of ActiveYou I, Dalen et al. [12] also applied cognitive interviews with children.

### 2.4. Data Analysis

The qualitative data from the cognitive interviews were coded directly in the audio files, and the thematic content analysis [26] was done with software support using MAXQDA [27]. Data were analyzed in relation to Tourangeau’s question-answer-model applying the four categories: comprehension, retrieval, judgment, and response [23]. After meaningful parts were identified, the essence of these sequences was extracted and labeled relevant categories. Comprehension, retrieval, judgment and response appeared to be the operating categories. Some examples are presented in Table 2 to give transparency to the analysis-process. As understanding or not understanding a concrete item, reflections on the meaning, or even providing suggestions for alternatives were the practical outcomes of the analysis, the results are presented as a whole. Thus, the items of ActiveYou II are presented following the order of the questionnaire.

## 3. Results

### 3.1. Characteristics of the Sample

Nine children participated in the cognitive interviews: two males and seven females, with a mean age of 12.6 years (SD = 1.1 years). Four had a physical disability, three had mild intellectual disability, and two had a complex disability. Five parents participated in the interviews to assist their children and give their perspectives.

### 3.2. Item Adjustments Based on the Cognitive Interviews

The analysis of the cognitive interviews showed several issues with the general phrasing of the questions or specific terminology, which lead to complications in the comprehension-phase of the question-answer-model. To clarify the results, Table 1 shows the items before and after the cognitive interviews. The formulations have been translated from Norwegian into English as close to the original as possible. The results are presented in the same order that they appear in the instrument, to keep the structure of the section close to the data collection procedure and the instrument.

Generally, it became clear that parents, interviewers, or both were needed to assist the children with intellectual disorders in answering the questions, especially when there were multiple alternatives to choose from. Regarding the frequency of attendance (item 1a/b), some children had problems finding the right category. When asking how often they did an activity, they answered “I do this every other Friday” or “I do this on Mondays and Thursdays.” They were more used to working with weekly schedules—as several parents explained—than thinking in quantitative categories like 1 or 2 times a month or 1 or 2 times a week. Some children did not understand the additional question Are you satisfied as it is? After explaining the intent of the question, the children suggested a formulation like “Is this as often as you like it?”, “Is it OK as it is?”, or “Do you like it as it is?”.

The second item on context (*With whom do you do the activity?*) also showed several problems regarding comprehension, specifically with specific terminology. For example, the children expressed difficulties with understanding or explaining the terms *relative* or *self-organized*. Participating parents suggested that *other adults* might be easier to understand than relatives for the children.

Regarding response alternatives, children and parents explained that children often did activities together with other children at school: not during lessons, but unorganized activities during breaks or before and after school using the school facilities. Therefore, they wished for another response alternative: “*with others at school*”. Similar to item 1b (*Are you satisfied as it is?),* children expressed their difficulties with item 2b (*Are you satisfied as it is?).* Here, participants suggested a formulation like “*Do you like it that way?*”.

Regarding the items on the subjective (unobservable component) sense of mastery (*How good do you think you can do the activity?*) and involvement/attraction (*Is it fun to do this activity*? *The activity is important to me*), children had no problems responding using the three-point Likert scale that consisted of a green (smiling), a yellow (neutral), and a red (negative) smiley face. However, children could not differentiate between the meaning/intent of the three items. When asking about their comprehension of why participation was important to them (regarding the question *Is the activity important to you?*), the children often answered, “*Because it is fun,*” which is much more an indicator for the question *Is it fun to do this activity?* When asked directly if they saw a difference between the three items, the majority of the children said “*No*” or could not explain what the difference might be. Some parents, who assisted their children, tried to explain that an activity might be important for them in order to train certain skills, improve their mobility, or improve their overall health. Therefore, it might be important even though they were not enjoying the activity that much. Still, this was not the children’s comprehension of the items.

Regarding the facilitating (item 6) and hindering factors (item 7), children were overwhelmed with the number of response alternatives. Again, children struggled with comprehension of different formulations and terminology. For example, *Activity available close by* was experienced as too vague. After explaining the meaning to the children, they suggested “I can do the activity where I live.” Furthermore, the formulation *The activity leader adapts the activity* was hard to comprehend for the children since both *activity leader* and *adapt* were unknown terms for the children. After explaining the meaning to the children, they suggested “The adults at the activity help me.” Regarding the financial costs of the activity (e.g., participation *for free* as a facilitator or *too expensive* as a barrier), children often had insufficient knowledge of the costs, especially if the costs might have been a barrier. This was something that only parents could relate to. Furthermore, children had problems with the formulation *I have the right equipment.* Here, children and parents suggested the alternative “I have everything I need”.

## 4. Discussion

### 4.1. Adjustments to the Instrument

The results of the cognitive interviews for ActiveYou II showed that adjustments were needed to make ActiveYou II more applicable for children with disabilities. The involvement of children from the target group was crucial in this developmental process. The children’s contribution was first and foremost connected to comprehension and response.

Many children felt it was difficult to deal with the number of response alternatives for the context, facilitators, and barriers. Even if they were able to make their judgment on the question, they had difficulties formatting their response into the given categories. This was based both on issues with terminology and the at times overwhelming number of answer alternatives. Therefore, it seemed appropriate to combine different alternatives or eliminate alternatives that did not seem relevant. Both the context and the facilitators and barriers need adjustments based on the children’s suggestions (as shown in Table 1). Gustafsson et al. [28], in their testing of the Swedish ICECAP-O, and Liljenquist et al. [25], during the development of the PES, reported the benefits of including cognitive interviews in a mixed-methods approach to instrument development and testing. Additionally, the development process of ActiveYou I benefited from the application of cognitive interviews [12]. Spencer, Bouffard, and Watkinson [17] argued that cognitive interviews were an important addition to the validation process of self-report instruments for children with disabilities. Experience from the current study supports this argument. This approach is in line with the Convention of the Rights of the Child [29] and the Convention on the Rights of Persons with Disabilities [30]. Incorporating these methods more often could help bring forward the perspective of a group that otherwise is too easily just talked about and not talked with. In a focus group study with persons with disabilities, Hammel et al. [31] found that the participants wanted to be consulted for their opinion about their participation.

Regarding the discussion of the items that measured the children’s level of involvement/attraction and sense of mastery, the cognitive interviews showed that the children had problems differentiating the intention/meaning of these items. Therefore, one of the three items (*The activity is important to* me) will be eliminated from the questionnaire. More in-depth research on the understanding of the remaining items (*How good do you think you can do the activity?* and *It is fun to do this activity*), possibly through additional interviews with children who also complete the digital version of the questionnaire, is needed to adjust the items to the target group further. Liljenquist et al. [25] showed how implementing cognitive interviews in multiple stages of the development and validation process improved the applicability of the final instrument for the target group. This is an approach that could also be beneficial for the development of ActiveYou II. When involving children and youth, the target age is important. The target group for ActiveYou II is age five to 17. However, the age of participants in the interviews ranged from 11 to 17. It is a limitation of the study that children 5 to 10 years of age were not included for practical reasons.

### 4.2. The Value of Cognitive Interviews in the Instrument Development Process

This study shows the value of including children from the target group in order to adjust the instrument to their needs. The results showed issues with comprehension of both questions and different response-alternatives. Like the situation when the children were able to find relevant information to answer the given question (retrieval) about frequency of participation, and formulate an answer, but could not transform their response to the given categories. Addressing these issues will most likely help to improve the validity of the new instrument. Many of these issues might not have been discovered without the application of cognitive interviews. Like Spencer, Bouffard, and Watkinson [17], the authors of this study think that these methods should be used more often when designing or adapting instruments for children and youth with disabilities.

### 4.3. Limitations

This study has some limitations. The main limitation might be the small sample and age range of the participating children. Since ActiveYou II is meant to serve a wide target group age 5 to 17 with different types of disabilities it would be an advantage to have participants with a broader age range for testing the instrument. Meanwhile, the sample at hand only covered children and youth aged 10 to 14. Still, with a variety of impairments, they represent a broad target group. The sample covered more girls than boys. Gender differences may, however, not be of great importance, when it comes to comprehension of wording of questions.

## 5. Conclusions

In conclusion, the current study showed how cognitive interviews with the target group can improve item quality and applicability of ActiveYou II. They identified the adjustment needed regarding unclear, difficult items on wording, formulations, amount, or different response alternatives. Furthermore, they provided new, adjusted or meaningful suggestions, and even suggested eliminated irrelevant items. In this way, cognitive interviews enhanced the development of ActiveYou II Therefore, this study can promote the approach of applying cognitive interviews–especially in combination with Tourangeau’s question-and-answer model–when designing instruments targeted at children and youth. Based on this study, the most relevant aspects of the question-answer-model are comprehension and response. Therefore, these two aspects should be considered carefully in the instrument-development process. Further new cognitive interviews could be useful in order to evaluate the effect of the adjustments done after this study. In addition, these interviews should include participants representing the whole age range of the target group of ActiveYou II.

At the same time, the results pointed out a direction for the further developmental process. Before ActiveYou II can be implemented, the psychometric proprieties need to be determined in future studies. This includes an adjustment to the recruitment of participants and appropriate methods for psychometric testing of the web-based instrument, such as test-retest reliability and internal consistency, in addition to construct validity and sensitivity to change.

## Figures and Tables

**Table 1:** The order of the items of ActiveYou II before and after the cognitive interviews.

Item	Cognitive Interviews	Adjustments for Further Development
**1a**	How often have did you do this activity during the last three month? ∙ 3–7 times a week ∙ 1 or 2 times a week ∙ 1 or 2 times a month ∙ Never	How often do you do this activity? ∙ 3–7 times a week ∙ 1 or 2 times a week ∙ 1 or 2 times a month ∙ Never
**1b**	Are you satisfied as it is? ∙ Yes ∙ No	Is this as often as you like it? ∙ Yes ∙ No
**2a**	With whom do you do the activity	
	∙ Alone ∙ With family (parents, siblings) ∙ With other relatives ∙ Sports club, community-center, youth-club ∙ Self-organized with friends ∙ With a personal assistant, leisure assistant…	∙ Alone ∙ With family ∙ With personal or leisure assistant ∙ With other adults ∙ Sports club, community-center, youth-club ∙ With friends ∙ Together with other kids at school
**2b**	Are you satisfied as it is?	Do you like it that way?
	∙ Yes ∙ No	∙ Yes ∙ No
**3**	How good do you think you can do the activity?	
	Smileys ∙ Negative ∙ Neutral ∙ Positive	Smileys ∙ Negative ∙ Neutral ∙ Positive
**4**	It is fun to do this activity.	
	Smileys ∙ Positive ∙ Neutral ∙ Negative	Smileys ∙ Negative ∙ Neutral ∙ Positive
**5**	The activity is important to me.	
	Smileys ∙ Positive ∙ Neutral ∙ Negative	
**6**	This makes it easier for me to participate	
	Somebody tells me where I can participate ∙ Activity available close by ∙ Participation is free ∙ Participate together with family ∙ Participate together with friends ∙ Mom, dad, or siblings assist me ∙ I have a personal assistant or leisure assistant ∙ The activity-leader adapts the activity ∙ I experience no pain ∙ I have the equipment I need	∙ I can participate together with my family ∙ I can participate together with friends ∙ Mom, dad, or siblings assist me ∙ I have a personal assistant or leisure assistant ∙ I have the equipment I need ∙ I can do the activity where I live ∙ Participation is free ∙ The other children at the activity are nice to me. ∙ The adults at the activity help me
7	This makes it difficult for me to participate	
	∙ I don’t know if there are possibilities to participate ∙ Activity not available where I live ∙ Too far away ∙ The date does not work for me ∙ Too expensive ∙ Nobody can assist me ∙ The others aren’t nice to me ∙ The activity-leader does not take care of me ∙ I’m too exhausted	∙ I don’t have the equipment I need ∙ Activity not available where I live ∙ The date does not work for me ∙ Too expensive ∙ Nobody can assist me ∙ The other children aren’t nice to me ∙ The adults at the activity do not help me ∙ I’m too exhausted ∙ I experience pain ∙ I feel insecure ∙ There is nothing that makes it difficult for me to participate

**Table 2:** Examples of the analysis process.

Original Quote	Extraction	Conceptual Theme
Interviewer: «How often do you go to the pool?» Child: «I go there every other Friday.» Interviewer: «Alright. What answer-alternative do you think you should cross than?» Barn: «I don’t know.»	Item 1a: The child is able to formulate an answer to the question but cannot translate the answer to the given categories in the questionnaire.	response
Interviewer: «Do you think there is a difference between these three questions?» (refer to items 3, 4 and 5) Child: «No.»	Items 3, 4, 5: The child cannot distinguish between the meaning of the items 3, 4 and 5	comprehension

## Data Availability

The data are not publicly available due to data-privacy regulations, but can be available on reasonable request.
